# The host immune response in respiratory virus infection: balancing virus clearance and immunopathology

**DOI:** 10.1007/s00281-016-0558-0

**Published:** 2016-03-10

**Authors:** Amy H. Newton, Amber Cardani, Thomas J. Braciale

**Affiliations:** 1 Beirne B. Carter Center for Immunology Research, University of Virginia, P.O. Box 801386, Charlottesville, VA 22908 USA; 2 Department of Pathology, University of Virginia, Charlottesville, VA USA; 3 Department of Microbiology, Immunology, and Cancer Biology, University of Virginia, Charlottesville, VA USA

**Keywords:** Lungs, Respiratory infection, Virus, Influenza, Inflammatory response

## Abstract

The respiratory tract is constantly exposed to the external environment, and therefore, must be equipped to respond to and eliminate pathogens. Viral clearance and resolution of infection requires a complex, multi-faceted response initiated by resident respiratory tract cells and innate immune cells and ultimately resolved by adaptive immune cells. Although an effective immune response to eliminate viral pathogens is essential, a prolonged or exaggerated response can damage the respiratory tract. Immune-mediated pulmonary damage is manifested clinically in a variety of ways depending on location and extent of injury. Thus, the antiviral immune response represents a balancing act between the elimination of virus and immune-mediated pulmonary injury. In this review, we highlight major components of the host response to acute viral infection and their role in contributing to mitigating respiratory damage. We also briefly describe common clinical manifestations of respiratory viral infection and morphological correlates. The continuing threat posed by pandemic influenza as well as the emergence of novel respiratory viruses also capable of producing severe acute lung injury such as SARS-CoV, MERS-CoV, and enterovirus D68, highlights the need for an understanding of the immune mechanisms that contribute to virus elimination and immune-mediated injury.

## Introduction

The cells that line the respiratory tract are continually exposed to the external environment, making the lungs a particularly vulnerable site for infection. Respiratory infections represent a major disease and economic burden worldwide. According to the CDC, influenza virus infection and associated complications are one of the top ten causes of death and result in millions of hospitalizations, costing over $10 billion each year in the USA [1]. Other respiratory virus such as highly pathogenic avian influenza and Severe Acute Respiratory Syndrome (SARS-CoV) and Middle Eastern Respiratory Syndrome (MERS-CoV) coronaviruses represents ever-present threats to human health globally. Therefore, understanding the factors, both virus-dependent and host-dependent, that regulate the development and severity of respiratory virus infections is critical for both the prevention and treatment of virus-associated disease in the respiratory tract.

A limited survey of respiratory viral infections reveals that respiratory viruses with distinct virion and genome structures, unique entry receptors, and modes of replication, cause similar clinical syndromes and sequelae (Table 1). These clinical observations and a growing body of experimental data suggest that the host response to infection rather than direct viral injury of respiratory cells primarily accounts for the clinical and pathologic changes observed during respiratory viral infections. This review, therefore, provides a brief overview of the contribution of host responses to lung pathology during primary acute virus infections rather than pathology caused directly by virus. A detailed, comprehensive comparison of the differences among respiratory viruses is not discussed here.

**Table 1:** Clinical presentation of respiratory viral infections

Virus	Entry receptor	Common symptoms	Clinical complications
Rhinovirus	ICAM-1 or LDL	Rhinorrhea, coryza, sneezing, sore throat, cough	Asymptomatic, mild to moderate upper-respiratory tract illness, bronchitis
Common coronavirus	Strain specific	Fever, rhinorrhea, coryza, sneezing, sore throat, cough	Mild to moderate upper-respiratory tract illness
Adenovirus	Strain specific penton	Fever, rhinorrhea, coryza, sneezing, sore throat, cough, pink eye, diarrhea, bladder infections	Mild to moderate upper-respiratory tract illness, croup, tonsilitis
Seasonal influenza	Sialic acids	Fever, rhinorrhea or stuffy nose, coryza, sore throat, cough, headache, myalgia	Mild to moderate upper-respiratory tract illness, bronchitis, croup
RSV	Nucleolin	Fever, rhinorrhea, coryza, sore throat, cough, wheezing, shortness of breath	Mild to moderate upper-respiratory tract illness, bronchitis, bronchiolitis, croup
Enterovirus D68	Sialic acids alpha2-6	Rhinorrhea, sneezing, cough, mouth blisters, myalgia; wheezing and dyspnea in more severe cases	Mild to moderate upper-respiratory tract illness, bronchitis, bronchiolitis, pneumonia
Pandemic influenza	Sialic acids	Fever, coryza, rhinorrhea or stuffy nose, sore throat, cough, headache, shortness of breath, dyspnea, myalgia	Bronchitis, croup, pneumonia, diffuse alveolar damage, acute respiratory distress syndrome, respiratory failure
SARS-CoV	ACE2	Fever, chills, cough, shortness of breath, dyspnea, myalgia	Rapidly progressive pneumonia, diffuse alveolar damage, severe acute respiratory distress syndrome, respiratory failure, fibrosis
MERS-CoV	CD26	Fever, chills or rigors, coryza, sore throat, non-productive cough, sputum production, shortness of breath, dyspnea, headache, vomiting, diarrhea, myalgia	Rapidly progressive pneumonia, diffuse alveolar damage, severe acute respiratory distress syndrome, respiratory failure, septic shock and multi-organ failure

## Direct viral injury

A virus must replicate and orchestrate the assembly of virion constituents to produce progeny virus and propagate itself. This often happens at the expense of the infected cell. A radical but ultimately effective response to stop virus replication is for the infected cell to self-destruct via apoptosis although some viruses have evolved strategies to circumvent this [2]. Cytopathology or death by starvation can also result from virus usurpation of host cellular machinery and metabolic processes [2]. Thus, death of infected cells caused directly by virus does play some role in lung pathology during infection. However, much of the clinical sequelae and damage to respiratory cells is a result of the host response to virus and virus-infected cells.

## Response of the host

### Viral sensing

Nearly all body cells have mechanisms to detect viruses (and other microbial pathogens) by pattern recognition receptors (PRRs) which recognize pathogen-associated molecular patterns (PAMPs) or molecules associated with viral and microbial pathogens but not typically found in host cells. PRRs that are important for detection of viruses include Toll-like receptors (TLRs), retinoic acid-inducible gene I (RIG-I), NOD-like receptors (NLRs), and other cytosolic virus sensors. Although these receptors are expressed in many types of cells, their activation in respiratory epithelial cells, typically among the first cell types to be infected, is critical in limiting virus spread and alerting the immune system to respond to the infection. Activation of PRRs in these cells by virus infection triggers production and release of type I and III interferons (IFNs) and other proinflammatory mediators (e.g., cytokines, chemokines, and antimicrobial peptides) which initiate the host innate and adaptive immune response. Thus, the degree of PRR activation throughout the respiratory tract ultimately influences the degree of immune cell recruitment and proinflammatory mediator release and subsequently, any immunopathology ensued.

### Interferon response

#### Type I IFNs (IFN-α and IFN-β)

Many different types of cellular sensors can detect viruses and induce the expression of the type I IFNs—IFN-α and IFN-β. Type I IFNs bind to the ubiquitously expressed IFNAR (IFN-α/β receptor), activating the JAK/STAT pathway [3]. The type I IFN response can induce the expression of hundreds of interferon stimulated genes (ISGs) which primarily serve to limit further virus spread and infection. However, type I IFNs can also directly activate immune cells (e.g., stimulating phagocytosis, dendritic cell maturation) as well as indirectly initiate immune responses (e.g., stimulating the production of chemokines and cytokines by respiratory cells). Intrinsic antiviral activity by type I IFNs includes impairing viral processes such as cell entry, replication, transcription, and translation [4]. Other ISG products can degrade viral nucleic acids or proteins, and many ISGs have yet to be fully characterized.

In addition to their intrinsic antiviral activity, type I IFNs promote the initiation of the adaptive immune response by mediating dendritic cell (DC) activation, enhancing effector functions of lymphocytes and macrophages, and stimulating the humoral (antibody) response to neutralize virus. Type I IFNs have a significant impact on DCs by (1) stimulating the differentiation and maturation of monocytes into DCs, (2) promoting migration of DCs to lymph nodes through induction of chemokine receptors, (3) enhancing antigen (Ag) presentation to CD4 T cells and cross-presentation to CD8 T cells, and (4) upregulating costimulatory molecules requisite for naïve T cell activation and effector T cell generation [3]. Thus, type I IFNs play an indirect role in orchestrating adaptive immune responses through the activation of DCs.

Type I IFNs directly enhance the functional activities of lymphocytes. They stimulate the production and secretion of IFN-γ (type II IFN) which in turn activates macrophages and phagocytosis, enhances Ag presentation by DCs, and directly limits viral replication [5]. Type I IFNs also augment cytotoxic activity of T cells and natural killer (NK) cells, thereby facilitating clearance of virus-infected cells and preventing further dissemination of the virus [5]. IFNs promote the humoral immune response indirectly through DC and T cell activation, but they can also directly activate B cells and promote a robust and effective virus-specific antibody response [6].

#### Type III IFNs (IL-28A/IFN-λ1, IL-28B/IFN-λ2, and IL-29/IFN-λ3)

Unlike IFNAR which is expressed in nearly all body cells, expression of the receptor for type III IFNs—IFNLR or interferon lambda receptor—and IFN-λ production is limited in the lungs primarily to epithelial cells [3]. Like type I IFNs, type III IFNs activate the JAK/STAT signaling pathway and thus, elicit similar intrinsic and extrinsic antiviral biological activities as described above for type I IFNs in airway epithelium [7, 8]. Interestingly, in response to viral stimuli, human airway epithelial cells produce greater amounts of IFN-λ than type I IFNs and thus, type III IFNs may be more effective against viral infection in respiratory cells than type I IFNs [8, 9].

As a consequence of their widespread effects on host immune responses, IFNs indirectly facilitate much of the inflammation and injury in the lungs during acute virus infection.

### Epithelial cells

Cytokines, chemokines, and other factors discussed in this section are also secreted by many types of immune cells (discussed later), and therefore will cause similar pathological or protective effects.

#### Cytokines

Airway epithelial cells secrete a host of cytokines, chemokines, antimicrobial peptides and other factors in response to viral infection. Cytokines beyond the IFNs produced by airway epithelium include interleukin-6 (IL-6), tumor necrosis factor-α (TNF-α), granulocyte colony stimulating factor (G-CSF), and granulocyte macrophage-CSF (GM-CSF). IL-6 and TNF-α are potent proinflammatory cytokines that modulate many types of immune cells. IL-6 facilitates the transition from the innate to adaptive immune response by driving down neutrophil activity while concurrently promoting the recruitment, differentiation, and activity of monocytes and T cells [10]. High IL-6 levels correlate with disease severity, but ablation of IL-6 signaling can lead to uncontrolled virus replication resulting in greater mortality [11]. TNF-α impairs viral replication, enhances cytotoxic activity, and cytokine production by leukocytes and activates endothelial cells [12]. Elevated levels of TNF-α have been associated with greater morbidity during infection with highly pathogenic virus, and blocking activity of TNF-α attenuates immune-mediated pathology [11].

G-CSF and GM-CSF both induce differentiation of myeloid lineage cells. G-CSF stimulates the production, differentiation, proliferation, and survival of neutrophils thereby mobilizing one of the first responders—neutrophils [13]. GM-CSF stimulates the proliferation and differentiation of various types of immune progenitor cells. In the lungs, GM-CSF induces the expansion and activation of pulmonary DCs and macrophages—immune responses necessary for an effective T cell response and viral clearance [14, 15]. Mice deficient in GM-CSF signaling are highly susceptible to respiratory viral infection, and exogenous delivery of GM-CSF is protective [15, 16].

#### Chemokines

Chemokines produced by respiratory epithelium stimulate the migration of both innate and adaptive immune cells to the lungs. IL-8/CXCL8 recruits neutrophils to the lungs, stimulates degranulation, and the subsequent release of cytotoxic and inflammatory mediators and may support neutrophil survival [17]. Elevated levels of IL-8 have been implicated in the pathogenesis of acute respiratory distress syndrome (ARDS) due to tissue damage likely caused by excessive release of neutrophil proteases and reactive oxygen species (ROS) [17].

IP-10/CXCL10 stimulates the chemotaxis of T cells, NK cells, and monocytes in addition to promoting monocyte and DC activation in concert with other cytokines. CXCL10/IP-10 may be protective in some cases since neutralization of IP-10 worsens disease caused by respiratory viral infection [18]. In other cases, CXCL10/IP-10 may contribute to extensive leukocyte recruitment, propagating a heightened inflammatory response and immune-mediated lung injury [19, 20].

CCL5/RANTES recruits several types of leukocytes: T cells, NK cells, monocytes, DCs, and granulocytes. In addition to its chemotactic properties, CCL5 can also activate T cells, monocytes, NK cells, and DCs promoting a diverse range of immune responses important in controlling virus infection and generating protective immunity [21]. In response to RSV (respiratory syncytial virus) infection, CCL5 may exacerbate inflammation and airway hyperreactivity [22], but deletion of CCL5 in response to influenza virus infection leads to decreased survival in mice [23]. Thus, the levels of CCL5 and type of viral pathogen may determine whether this chemokine is beneficial or detrimental to the host during infection. Respiratory epithelium also produces several other chemokines whose role in protecting or contributing to pulmonary pathology is not entirely clear. While most chemokines released by respiratory cells in response to PRRs, IFNs, and proinflammatory cytokines are not inherently pathogenic in of themselves, redundant or concerted chemotaxis and/or substantial amounts of chemokines may induce excessive leukocyte infiltration and activity, damaging respiratory epithelium.

#### Antimicrobial products and other secreted factors

Respiratory epithelial cells secrete numerous broad-spectrum antimicrobial/antiviral products that directly inhibit respiratory pathogens and in some cases, modulate immune responses. The most abundant antimicrobial factors in the lungs are lysozyme, lactoferrin, and secretory leukocyte proteinase inhibitor (SLPI) [24]. Lactoferrin, a glycoprotein secreted by mucosal epithelium, can prevent both DNA and RNA viruses from infecting cells by directly binding virus and blocking host receptors used by viruses to gain entry into cells [25]. Lysozyme and SLPI while effective against some microbial pathogens have limited antiviral capabilities in the lungs. However, SLPI plays a major role in protecting mucosal epithelium from damaging proteolytic enzymes released by epithelia and inflammatory cells, thus, limiting lung injury and promoting wound healing [26].

Airway epithelial cells produce substantial amounts of β-defensins—cationic peptides that exert both antimicrobial and antiviral properties [24]. β-defensins neutralize viruses, induce cytokine production by epithelial cells, and recruit immature DCs and memory T cells [24]. Cathelicidins (LL37) also exert broad-spectrum antimicrobial properties and can disrupt the envelopes of viruses, recruit immune cells, and enhance chemokine and cytokine production from local cells, promoting inflammatory responses [27].

Respiratory epithelial cells constitutively produce nitric oxide (NO) which is further increased by exposure to proinflammatory cytokines and respiratory viruses [28]. NO and products formed by NO activity inhibit viral proteins, transcription and replication [29]. The indiscriminate activity of NO can also modify host cellular proteins and induce generation of toxic cellular species such as free radicals and reactive nitrogen intermediates that damage pulmonary epithelium [29, 30].

Alveolar type II epithelial cells constitutively produce and secrete four surfactant proteins (SP-A, −B, −C, and −D). SP-B and SP-C contribute to the properties of surfactant which reduces the surface tension in the alveoli; thus, SP-B and SP-C serve a critical role in pulmonary function but have limited antiviral activity [30]. Hydrophilic SP-A and SP-D function as collectins—secreted, soluble PRRs that recognize many viral and microbial PAMPs and enhance opsonization of pathogens. Targeting viruses by these collectins facilitates phagocytosis and clearance by macrophages and neutrophils [31].

### Endothelial cells

Although respiratory epithelial cells are the primary targets of most respiratory viruses, infection of endothelium by virus can occur but is highly dependent on the viral pathogen and frequently is species-specific. For example, human RSV productively infects pulmonary vascular endothelium leading to endothelial cell activation but also cell death [32]. In contrast, influenza viruses generally do not infect human endothelium although some effectively replicate within avian endothelium [33]. Therefore, the degree to which endothelial cells contribute to respiratory pathogenesis depends in part on virus tropism and the direct cytopathic effects and cytokine signaling induced by a replicating virus (similar to virus-infected respiratory epithelium). Viruses that infect endothelium may induce more significant lung injury particularly to the alveolar epithelium as a result of increased vascular permeability, edema, and spatial proximity to alveolar cells. Hantavirus, unlike most respiratory viruses, primarily targets microvascular endothelium causing significant microvascular leakage and pulmonary edema [34]; notably, hantavirus pulmonary syndrome—characterized by the filling of the lungs with fluid—has a mortality rate of 38 %.

Release of proinflammatory cytokines, NO, and ROS from epithelium and leukocytes can enhance endothelial permeability and disrupt the integrity of the endothelial-epithelial barrier [35]. Damage to alveolar epithelium can expose and increase the susceptibility of localized endothelium to injury. Increased vascular permeability and pulmonary edema impairs efficient gas exchange between the airways and blood, resulting in respiratory distress. Thus, endothelium damage caused directly by virus or indirectly by inflammatory processes can play a significant role in pulmonary injury in response to respiratory infection.

Regardless of infectivity of endothelium, endothelial cells play an essential role in leukocyte migration and can modulate the immune response, particularly early innate immune responses to virus infection. Extravasation of immune cells into the lungs from the circulation (or alternatively resident pulmonary DC migration to the draining lymph nodes) is highly dependent on interactions between endothelium and leukocytes. Selectins and adhesion molecules requisite for extravasation are upregulated on endothelium in response to cytokines and growth factors secreted by respiratory epithelium and other neighboring cells [35]. Activated endothelium can also secrete proinflammatory cytokines and chemokines such as IL-6, MIG/CXCL9, IP-10/CXCL10, type I and type II IFNs, MCP-1/CCL2, and TNF-α in response to a virus infection [35]. Inhibition of these processes in endothelial cells in an animal model of lethal influenza infection greatly improved survival [36]. Thus, endothelial cells can facilitate pulmonary injury by heightened leukocyte recruitment and cytokine secretion during a highly pathogenic virus infection. The extent of pulmonary tissue that is infected or damaged and the amounts of proinflammatory cytokines and chemokines generated in response, influences the degree of endothelial activation. Ultimately, if uncontrolled or too extensive, these processes can be more damaging than beneficial by recruiting more immune cells that either injure the lungs directly or further propagate inflammation causing a “cytokine storm.” However, insufficient leukocyte recruitment or cytokine production can leave the host susceptible to the virus.

### Alveolar macrophages

Alveolar macrophages—the tissue-resident macrophages of the lungs—are localized to the airspaces within alveoli, uniquely positioning them to respond to threats to the lower airways. During homeostasis, alveolar macrophages regularly encounter innocuous antigens. To prevent inappropriate inflammatory responses to these benign antigens, the lung environment maintains alveolar macrophages in a suppressive state. Negative regulation of alveolar macrophages is accomplished through expression of IL-10, alphaV/beta6 integrin, GM-CSF, CD200 receptor, and pulmonary surfactants by alveolar epithelium [37]. Upon respiratory virus infection, the environment of the alveolar sac quickly changes, and expression of the negative regulators declines. This change in the lung environment coupled with detection of viral antigens and exposure to proinflammatory mediators enables alveolar macrophages to adopt a proinflammatory phenotype, which assists in initiating the host immune response and viral clearance. While the mechanism of protection remains unclear, a growing body of evidence has demonstrated that alveolar macrophages are essential during respiratory viral infection. For example, during RSV infection, they are responsible for early cytokine and IFN production that orchestrates the initial antiviral response [38].

### Neutrophils

Among the first immune cells to arrive to the site of infection, neutrophils excel in eliminating infected cells and clearing microbial pathogens, dead cells, and debris. Neutrophils are generally not productively infected by respiratory viruses but do phagocytose virions, viral particles, and apoptotic bodies containing virus [39]. Once phagocytosed, neutrophils utilize a host of proteolytic enzymes and antimicrobial peptides released from intracellular granules and ROS produced by NAPDH oxidase to kill or inactivate pathogens [40]. Neutrophil granules and their contents may also be secreted and released extracellularly. Some of the antimicrobial peptides secreted by neutrophils include defensins, lactoferrin, lysozyme, and cathelicidins (LL-37). In addition to these mechanisms, neutrophils can form and release neutrophil extracellular traps (NETs)—molecular traps composed of decondensed chromatin, histones, proteases, and antimicrobial proteins [41]. The release of NETs immobilizes pathogens and prevents further dissemination.

Sufficient neutrophil recruitment and activity control virus dissemination and mitigate severe disease. However, an excessive neutrophil response can be detrimental to the host by causing uncontrolled inflammation and indiscriminate damage to respiratory epithelium [39]. ROS, hydrolytic enzymes and myeloperoxidase released into the extracellular space can injury alveolar epithelium and endothelium [35]. A genetic deficiency in NAPDH oxidase—the enzyme responsible for ROS production and implicated in NET formation—attenuates lung injury and improves recovery from influenza virus infection in mice [42]. NETs may also damage respiratory epithelium and endothelium possibly by exposure to hydrolytic enzymes and cytotoxic proteins [35].

In addition to their phagocytic and cytotoxic activities, neutrophils can mediate both innate and adaptive immune processes. Neutrophils secrete chemokines that can recruit more neutrophils to the site of inflammation; elevated levels of these chemokines have been associated with more severe lung pathology during infection [17, 19, 20, 43]. Normally neutrophils undergo apoptosis, NETosis (programmed death that results in the formation of NETs), or some other form of cellular death and therefore, inflammatory and cytotoxic activity of this non-specific, potent granulocyte is typically short-lived. Apoptotic bodies and debris left by apoptotic neutrophils is then cleared by macrophages. However, if programmed cellular death of neutrophils or clearance by macrophages is impaired, both respiratory and immune cells may be exposed to damaging or activating factors propagating severe inflammation and acute lung injury [44].

Although historically the role of neutrophils in the immune response has been centered on inflammatory and innate responses, novel roles for neutrophils in shaping the adaptive immune response have emerged. Some neutrophils can express B cell-activating cytokines that support B cell activity and may function as “B-cell helper” neutrophils [40]. Neutrophils can suppress T cell responses through the production of suppressive factors such as inducible nitric oxide synthase (iNOS) and arginase 1. In other cases, neutrophils may promote T cell responses through the secretion of IFN-γ or Ag presentation [40, 45]. The mechanisms that neutrophils utilize to support adaptive immune responses during respiratory viral infections and the impact that these activities have on viral clearance, injury and recovery will need to be investigated further.

### Innate lymphocytes

#### NK cells

Natural killer (NK) cells are part of a family of innate lymphoid cells (ILC) that includes ILC1, −2 and −3. The role of ILC (other than NK cells) during respiratory virus infection is unclear although ILC2 have been reported to facilitate repair after lung injury [46]. NK cells respond to viral infection within a few days producing significant amounts of IFN-γ, killing virus-infected cells, and supporting the adaptive immune response. NK cells are uniquely equipped with a diverse set of receptors that enable them to distinguish normal cells (via inhibitory receptors) from virus-infected or transformed cells (via activating receptors). Activating receptors can recognize viral or tumor antigens—allowing the NK cells to eliminate both infected and tumor cells. In response to chemokines, additional NK cells are recruited within a few days (following neutrophil recruitment but prior to the arrival of effector T cells) and facilitate the transition from the initial, potent inflammatory responses to the more specific adaptive response [47].

Activation of NK cells triggers cytotoxic activity which eliminates virus-infected cells. NK cells kill infected cells by releasing cytotoxic granules, engaging death receptors, or utilizing antibody-dependent cell-mediated cytotoxicity (ADCC)—a process in which antibodies bind viral proteins expressed on the surface of infected cells marking them for elimination [47, 48]. All of these cytotoxic processes promote viral clearance and limit viral spread. However, viruses such as some strains of influenza virus have adapted mechanisms to activate inhibitory receptor signaling, preventing NK-mediated killing [47]. Additionally, despite inefficient viral replication within NK cells, infection of NK cells by some respiratory viruses can induce apoptosis limiting the numbers of NK cells and thus, early cytotoxic functions to control viral dissemination [39]. Depletion of pulmonary NK cells impairs viral clearance and subsequently, leads to more severe disease [47, 49]. NK cells also augment CTL (cytotoxic T lymphocyte) activity through IFN-γ production [49, 50]. In some viral infections such as RSV, robust production of IFN-γ by NK cells may contribute to lung injury [51]. Therefore, the type of virus and inoculating dose may influence whether NK cells contribute to lung pathology or not. Finally, NK cells have recently been shown to secrete IL-22 which promotes tissue repair mechanisms [52]. Overall, NK cells appear to primarily have a beneficial role during respiratory viral infection by clearing virus, promoting the CTL response, and facilitating tissue repair.

### Unconventional T cells

#### NKT cells

Natural killer T cells (NKTs) represent a heterogeneous population of T cells that possess properties of both NK and T cells. NKTs may be classified as either type I (or invariant NKT cells) or type II NKT cells depending on the type of TCR expressed [53]. The best-characterized and most studied subset of NKTs in the context of viral infections is invariant NKT (iNKT) cells which express a semivariant TCR that recognizes lipid molecules—most likely endogenous self lipids during viral infection since viruses lack exogenous lipid antigens. Cytokines may also activate iNKTs during respiratory viral infection. iNKTs support effector responses but also limit the degree of lung injury by regulating other immune responses and virus-mediated sequelae. iNKTs support CTL and NK responses through cytokine production and promoting the licensing, cross-priming and maturation of DCs, and limiting the immunosuppressive activity of virally induced myeloid-derived suppressor cells (MDSCs) [53, 54]. iNKTs limit inflammation-mediated damage by regulating inflammatory monocyte activity and promote repair processes during the recovery phase through the secretion of IL-22 [54]. Mice deficient in iNKTs suffer from more severe disease in response to RSV or influenza virus infection [54]. iNKT cells exhibit multi-faceted roles in modulating the immune response to viral pathogens and appear to be mostly beneficial in limiting lung injury during acute infection.

#### γδ T cells

In contrast to αβ T cells, γδ T cells express TCRs of limited diversity composed of γδ chains instead of αβ chains and predominantly reside within mucosal or non-lymphoid tissues such as the skin, lungs, and intestine [55]. Because γδ T cells are mostly tissue-resident cells, it has been suggested that they may serve an important role in the initial response and defense against pathogens [55]. In the lungs, γδ T cells can suppress inflammation and help regulate other immune responses to mitigate severe lung injury and promote tissue repair [55, 56]. In patients that survived SARS-CoV infection, expansion of an effector memory γδ T cell population capable of producing IFN-γ and directly killing SARS-CoV-infected cells may have protected them from succumbing to SARS [56]. In other cases, γδ T cells promote inflammatory processes by secreting proinflammatory cytokines and chemokines, contributing to more severe disease [57]. In response to lung injury, γδ T cells promote tissue repair and protect against pulmonary fibrosis through the secretion of IL-22 and suppression of other immune cells [55, 58]. Contradicting functional roles for γδ T cells during respiratory infection might be attributed to different viruses, the functions of certain subsets of γδ T cells, or different activity by γδ T cells during the various phases of infection.

### Dendritic cells

Dendritic cells (DCs) represent a diverse population of cells that serve as fundamental orchestrators of the immune response. DCs activate T cells, secrete various cytokines and chemokines, and promote protective adaptive immunity. Some DC subsets preferentially reside in either lymphoid or non-lymphoid tissue; while other subsets can migrate between these tissues. Under normal, non-inflamed conditions, respiratory DC populations can be found below and within conducting airway epithelium as well as in the lower airways within the walls of alveoli [59]. Thus, they are strategically positioned to sample antigens throughout the upper and lower respiratory tract. In mice, resident pulmonary DCs include conventional CD11b+ DCs and CD103+ DCs as well as plasmacytoid DCs (pDCs) [59]. Human pulmonary DC populations similarly include pDCs and two subsets of myeloid DCs that functionally resemble the CD11b+ (CD1+ DCs) and CD103+ (CD141+/CLEC 9A DCs) subsets found in mice [39]. In response to infection, some CD103+ and CD11b+ DCs acquire viral antigens and migrate to the draining lymph nodes (DLN) activating adaptive immune responses while other DCs remain in the lungs promoting local immune responses [59, 60]. Nonresident monocyte-derived DCs (mo-DCs) also accumulate in the lungs as inflammatory monocytes migrate into the lungs and differentiate into either macrophages or DCs. DCs can be activated directly by virus through PRRs and indirectly by proinflammatory chemokines and cytokines released by respiratory epithelium and other resident immune cells [59].

#### Conventional DCs

In the lungs, CD103+ DCs are mostly associated with the mucosal epithelium of the conducting airways and function primarily as APCs, activating both CD8 and CD4 T cell proliferation and differentiation through antigen presentation [59, 61]. CD103+ DCs migrate to the DLN within 2–4 days following infection where they efficiently activate both CD4 and CD8 naïve and memory T cells that recognize viral antigens [63, 64]. CD103+ DCs are the most robust stimulators of naïve CD8 T cell activation and elicit protective CD8 immunity because they express receptors for uptake of apoptotic bodies containing virus and efficiently cross-present exogenously acquired antigens [59–61]. Both CD103+ and CD11b+ DCs activate naïve CD4 T cells, drive T helper 1 (Th1) responses, and generate effective memory T cell populations to protect against subsequent infections [61]. CD11b+ DCs are also capable of presenting antigen and activating both CD4 and CD8 T cell responses to control respiratory viral infection but differ in a few distinct ways from the CD103+ DC population. CD11b+ DCs arrive a bit later to the draining lymph nodes (5–7 days after infection) where they may promote expansion of previously activated effector CD8 T cells and secrete robust amounts of proinflammatory chemokines [59, 61].

#### pDCs

pDCs are potent producers of type I IFNs during respiratory viral infection, and thus play a role in the initial sensing of viral pathogens and initiating inflammatory and innate immune responses. Unexpectedly, in response to influenza virus, the absence of pDCs had no effect on viral clearance or disease severity [62]. Although pDCs can transport viral antigens from the lungs to the DLN, pDCs are poor stimulators of naïve T cell activation and differentiation due to low expression of costimulatory molecules [59, 61]. Thus, pDCs may be dispensable during certain respiratory viral infections. In response to RSV infection, however, pDCs may suppress inflammation and promote viral clearance, protecting against severe lung injury [62]. The precise functions of pDCs during respiratory viral infections warrant further study to understand how different subsets of pDCs in response to different viruses contribute to viral clearance, modulation of the immune response, and lung injury.

#### Monocyte-derived DCs

A large fraction of mo-DCs initially arrive in the lungs as “monocytes” which then differentiate into DCs in response to type I IFNs and direct infection with virus [59, 60]. These mo-DCs can support naïve CD8 T cell activation, Th1 differentiation and cytotoxic effector functions [59]. They can also further propagate an inflammatory response through the production of CXCL10/IP-10 and CCL2/MCP-1, recruiting more inflammatory monocytes to the lungs. During a recall response, mo-DCs may activate cytotoxic responses of memory CD8 T cells and NK cells through cytokine production, enabling swift and efficient pathogen clearance [60]. Elevated numbers of mo-DCs that express CCR2 (chemokine receptor for CCL2) or produce high amounts of TNF-α and NO are associated with greater morbidity and mortality in response to viral infection [11, 59, 60]. While blocking mo-DC activity or deficiency in CCR2 improves survival and mitigates severe lung pathology in experimental virus infection, complete depletion of mo-DCs leads to uncontrolled virus spread and more severe disease [11, 59]. Thus, a balanced response by DCs that promotes viral clearance and effector responses without eliciting too much inflammation is probably the key in controlling virus- or immune-mediated lung injury.

### Inflammatory monocytes

Inflammatory monocytes are recruited from the circulation to the lungs following viral infection. They produce type I IFNs and chemokines and can differentiate into DCs (as discussed above) or macrophages which promote viral clearance, cytotoxic activity, and T cell activation [63]. Potentially, a defect in inflammatory monocyte activity or recruitment could impact any of these immune processes. Since inflammatory monocytes depend on CCR2 to migrate to the lungs, a deficiency in this chemokine receptor impairs the recruitment of inflammatory monocytes (and the subsequent populations derived from them) which results in improved survival and diminished lung pathology [11, 59]. However, a defect in the CD8+ T cell response and viral clearance may also occur when the CCR2+ population is completely eliminated. An attenuated rather than complete deletion of the inflammatory monocyte response has been shown to allow effective viral clearance while limiting inflammatory and immune-mediated damage to the lungs [11, 63]. Similarly to mo-DCs, the role of inflammatory monocytes in lung pathology may be detrimental or beneficial depending on the degree of the response and viral pathogen.

### Adaptive immune responses

Although the innate immune response plays a significant role in initiating both antiviral responses and adaptive immune responses, ultimately the adaptive immune response is responsible for complete viral clearance by halting viral replication to prevent the generation of new virions and eliminating infectious virions. Because adaptive immune cells are dependent on early innate immune responses, this does not, however, mitigate the role of innate immune response to virus infection.

#### Humoral immune response: B cells and CD4 helper T cells

B cells contribute to virus clearance predominantly through production of virus-specific antibodies which can (1) neutralize, opsonize, and inactivate virions or (2) initiate killing of infected cells. Preventing the spread of infectious virions from infected cells to neighboring cells is essential to controlling virus dissemination. Neutralizing antibodies produced by B cells effectively prevent free virions from invading uninfected cells by blocking surface proteins on the virus that bind to host receptors for cellular entry. Viruses coated in antibodies are also tagged for inactivation by complement proteins or elimination by phagocytic immune cells such as macrophages and neutrophils. Antibodies can bind to viral proteins expressed on the surface of infected cells, triggering the complement cascade and antibody-mediated cell-mediated cytotoxicity (ADCC)—processes that ultimately eliminate infected cells. Although essential to viral clearance, these cytotoxic processes if extensive can compromise respiratory function through the loss of airway epithelium, disruption of the epithelial-endothelial barrier, and accumulation of apoptotic bodies and cellular debris in the airways.

A subset of subset of CD4 T cells called follicular helper T (T_FH_) cells play a critical role in facilitating an effective B cell response during infection. Adoptively transferred CD4 T cells protect athymic mice (mice that lack functional T cells) from succumbing to influenza infection. However, when CD4 T cells are adoptively transferred into SCID (severe combined immunodeficiency) mice that lack functional T and B cells, CD4 T cells are no longer sufficient for protection, and depletion of CD4 T cells during SARS-CoV infection coincided with a reduction in antibody production [64]. During infection, T_FH_ cells promote the formation of germinal centers in secondary lymphoid tissues where virus-specific B cells mature, proliferate, undergo antibody class switching, and differentiate into either antibody-producing plasma cells or long-term memory cells. T_FH_ cell numbers have been shown to correlate with the production of influenza-specific IgG and IgM antibodies after vaccination in healthy young adults [65].

Antibody-mediated protection is the goal of many vaccine strategies because of its critical role in viral clearance. Each year influenza vaccines are administered in order to enrich circulating neutralizing antibodies specific to that year’s predicted strains. This strategy has been substantiated by data demonstrating that rhinovirus and influenza-neutralizing antibodies in the serum, prior to infection, correlate with disease protection [66]. Similarly, a neutralizing antibody response to the spike glycoprotein of SARS-CoV has also been shown to be completely protective for a susceptible host although antibody titers are relatively short lived [67]. One of the disadvantages to a vaccine that primarily elicits an antibody response, though, is the lack of a protective, cell-mediated immune response which is particularly important in the context of viral infections.

#### Cell-mediated immune response: CD8+ T cells

Clearance of intracellular pathogens, such as viruses, requires elimination of the infected cells. Athymic mice which produce mature T cells (but do have an intact antibody response), do not recover from influenza infection due to the inability to clear virus. SARS-CoV are relatively new pulmonary viruses whose pathogeneses are still being elucidated; however, research indicates that CD8 T cells are essential for virus clearance. Adoptive transfer of SARS-CoV-activated T cells into SCID mice enhanced survival and reduced pulmonary virus titers [68]. Thus, the elimination of virus-infected cells through cell-mediated immunity is important to completely resolve infection. The primary function of CD8 T cells during respiratory viral infections is the elimination of virus-infected cells by inducing apoptosis through a variety of mechanisms. CD8 T cells engage death receptors such as FAS expressed by virus-infected cells and release perforin and granzymes which create pores in the cell membrane and initiate apoptotic pathways, respectively.

While essential, adaptive immune-mediated clearance of virus-infected cells can compromise the respiratory tract, and if unregulated, will cause catastrophic injury to the host tissue. This has been demonstrated in multiple models of adaptive immune deficient mice. Despite not being able to clear virus, RAG1 (recombination-activation gene-1) knockout mice that lack both functional T and B cells have delayed morbidity and mortality after high dose inoculation with influenza virus [69]. Furthermore, virus-specific CD8 T cells in RAG1 knockout mice exacerbate lung injury and accelerate mortality [69]. During experimental RSV infection, depletion of both CD4 and CD8 T cells enabled continued virus replication, but no illness was evident [70]. Ultimately, the adaptive immune response is required for resolution of infection and confers long-term protection from future encounters with the virus, preventing future disease; however, the adaptive immune response is also very potent and if extensive, can cause sufficient respiratory damage to impair pulmonary function.

### Immunopathology

Normal respiratory function is dependent on the preservation of pulmonary architecture and the endothelial-epithelial barrier between the airways and circulation. During viral infection, pulmonary architecture is frequently compromised primarily by the host immune response and to a lesser extent directly by virus replication in most cases. The extent of structural alteration, functional compromise and subsequently, clinical manifestations of infection are dependent on many factors but most prominently on the degree of virus dissemination throughout the respiratory tract.

### Infection of the upper respiratory tract (URT)

Often the URT is the initial site of viral replication since many respiratory viruses are inhaled or transferred by contact to the nasal mucosa. In healthy patients without pre-existing conditions, infections with common strains of rhinovirus, coronavirus, and adenovirus are typically limited to the upper airways. Symptomatic viral infection of the URT (coryza, rhinorrhea, cough, and sore throat) (Table 1) reflects loss of cellular tight junctions, vascular leakage and edema, increased mucus production, and apoptosis, necrosis, and sloughing of epithelial cells [71–73]. Recruitment of neutrophils and mononuclear cells into the URT further propagates edema and hyper-secretion of mucus, exacerbating nasal congestion, sneezing, and coughing in patients [71–73].

### Bronchitis and bronchiolitis

Viruses capable of producing more severe infections such as influenza, proceed from the URT to the LRT where they disseminate to the bronchi and bronchioles and if not controlled, alveoli. Patient monitoring during respiratory viral infection corroborates this, as upper respiratory illness precedes any LRT (lower respiratory tract) symptoms. Among respiratory viruses, influenza viruses, SARS-CoV, and MERS-CoV are commonly capable of spreading into the bronchi and bronchioles in otherwise healthy individuals. However, in infants and young children, RSV frequently infects small conducting airways and is a leading cause of pediatric hospitalization worldwide [74]. Enterovirus d68 is also capable of rapid progression to the conducting airways and gained particular notoriety in 2014 after an outbreak in the USA resulted in significant pediatric hospitalization with severe respiratory compromise and increased mortality.

Infection of the bronchi and bronchioles leads to similar cellular changes and localized inflammation as in URT infections. However, because of the progressively decreasing diameter of LRT conducting airways, inflammation and injury in this compartment—e.g., desquamation of airway epithelial cells in response to RSV or airway epithelial cell hyperplasia during influenza infection—results in more severe compromise of respiratory function. The absence of ciliated epithelial cells in the LRT limits the expulsion of mucus, proteinaceous fluid, bacteria, and cellular fragments from these airways. This is compounded by enhanced epithelial mucus secretion, vasculature leak, edema, mucosal inflammation, and epithelial sloughing. The influx of inflammatory cells in response to signals from infected epithelium and tissue resident innate immune cells increases edema, vascular congestion, and tissue swelling further limiting air passage through smaller airways. The buildup of edematous fluid, mucus and fibrin plugs in small bronchi and bronchioles can restrict airflow to terminal airways further preventing efficient gas exchange. Increased pressure within the blood vessels leaks more fluid into the airspaces. As all these factors build up in the already restricted bronchi and bronchioles, they begin to clot and form plugs that prevent gas exchange. This manifests clinically as wheezing and shortness of breath. In serious cases, this can lead to difficulty in breathing which requires oxygen therapy. Typically with supportive care, bronchitis and bronchiolitis will resolve in a few days without lasting effects.

### Pneumonia

While viruses such as RSV and Enterovirus D68 are capable of reaching the terminal airways, influenza viruses and the SARS-CoV and MERS-CoV coronaviruses more frequently reach the terminal airways, and thus are more likely to disrupt pulmonary function and cause pneumonia [72]. The terminal airways are the site of gas exchange and make up the majority of the airway surface of the lung. During infection, loss of tight junctions, vasculature leak, buildup of edematous fluid and fibrin in the alveolar airspaces can result in necrosis of the alveolar cells and hyaline membrane formation. If severe, these changes can lead to diffuse alveolar damage (DAD), manifested clinically as ARDS and respiratory failure. Inflammatory processes and immune responses as described previously compromise the function and properties of terminal airways and terminal airway epithelial cells in a fashion analogous to that of conducting airways. Furthermore, a loss of surface tension and surfactant resulting in alveolar collapse represent additional threats to pulmonary function in the terminal airways. The terminal airways of survivors of acute viral pneumonia can result in alteration of pulmonary elasticity due to organization of inflammatory processes during the resolution phase which may have lasting consequences and put the host at risk for superinfections [72, 73, 75]. Although not discussed here, bacterial superinfection or co-infection are important sequelae of virus infection in the LRT particularly during influenza virus infection.

## Conclusions

Not surprisingly, the immunopathology of respiratory virus infection reflects a complex interplay involving direct effects by a given virus and other viral factors but also the response of resident respiratory cells and recruited innate and adaptive immune cells to the lungs. Studies carried out over the last several decades suggest that in most—but not all—instances, the host response to respiratory virus infection (rather than the direct effects of virus replication) dictates the type and extent of injury incurred.
